# Clinical characteristics and antimicrobial management of invasive *Mycoplasma hominis* infection: a case report and literature review

**DOI:** 10.3389/fmed.2026.1728188

**Published:** 2026-02-16

**Authors:** Yanhua Liu, Fuxing Li, Jinyan Xie, Longhua Hu, Shumin Gu, Yunwei Zheng, Xingwei Cao, Yaping Hang, Yanping Xiao, Shan Zou, Qiaoshi Zhong, Yanhui Chen

**Affiliations:** 1 Jiangxi Province Key Laboratory of Immunology and Inflammation, Jiangxi Provincial Clinical Research Center for Laboratory Medicine, Department of Clinical Laboratory, The Second Affiliated Hospital, Jiangxi Medical College, Nanchang University, Nanchang, Jiangxi, China; 2 Department of Clinical Laboratory, Hunan University of Medicine General Hospital, Huaihua, Hunan, China; 3 Jiangxi Province Key Laboratory of Molecular Medicine, The Second Affiliated Hospital, Jiangxi Medical College, Nanchang University, Nanchang, Jiangxi, China

**Keywords:** antimicrobial therapy, clinical characteristics, doxycycline, fluoroquinolones, *Mycoplasma hominis* (*M. hominis*)

## Abstract

**Objective:**

This research aims to report a case of invasive *Mycoplasma hominis* (*M. hominis*) infection of a distal fibular wound and to provide a review of the literature in order to elucidate the clinical features and antimicrobial management of this uncommon pathogen.

**Methods:**

We describe a case of invasive *M. hominis* wound infection in a 60-year-old male following an open distal fibular fracture. In addition, we performed a narrative review of the literature via a PubMed search for reports of invasive *M. hominis* infection in adults published up to August 2025, restricted to adult cases. Extracted variables included demographic characteristics, underlying conditions, infection sites, antimicrobial regimens, and clinical outcomes.

**Results:**

Invasive *M. hominis* infection was most common in patients with surgery/urinary catheterization history, cardiopulmonary insufficiency, immunosuppression, or post-transplantation status. Primary infection sites were skin/bone, pleural/peritoneal effusions, and the central nervous system. Among 65 cases, 17 (26.2%) experienced treatment failure. Failure rates were 22.5% (9/40) in doxycycline-containing regimens and 32.0% (8/25) in non-doxycycline regimens, with no significant difference (*p* = 0.397). Prolonged antimicrobial therapy was protective against failure [OR 0.089 (0.017–0.466)], while post-transplantation status increased risk [OR 6.045 (1.053–34.710)]. Mortality was often multifactorial, especially in transplant recipients.

**Conclusion:**

Invasive *M. hominis* infections, though likely underdiagnosed and underreported, can lead to severe outcomes, particularly in patients with a history of surgery, urinary catheterization, or immunosuppression. Prolonged antimicrobial therapy is associated with improved outcomes, whereas post-transplantation status increases the risk of failure. Doxycycline-containing regimens, especially combined with fluoroquinolones, are a preferred therapeutic strategy. Heightened clinical vigilance and optimized diagnostic and therapeutic approaches are critical to reduce treatment failure.

## Introduction

*Mycoplasma hominis* (*M. hominis*) is a facultative anaerobic bacterium that lacks a peptidoglycan cell wall. This unique characteristic renders it undetectable by conventional Gram or acid-fast staining and intrinsically resistant to *β*-lactam antibiotics (1, 2). *Mycoplasma hominis* commonly colonizes the human genitourinary tract, with reported rates of 21%–54% in healthy adults (3), and is primarily associated with genitourinary infections such as pelvic inflammatory disease and bacterial vaginosis (4, 5).

In contrast, extragenital infections due to *M. hominis* are considered uncommon but can lead to severe invasive diseases, including postoperative wound infections, mediastinitis, and central nervous system involvement, with reported mortality rates ranging from 10% to 54% (6, 7). Diagnosis is particularly challenging because the organism grows slowly, requires specific culture conditions, and is easily overlooked in routine microbiological workflows (1–7). Consequently, infections are often missed or delayed, complicating clinical management. Furthermore, its intrinsic resistance profile narrows the spectrum of effective antimicrobials, making the choice of empiric therapy difficult. While individual case reports and small series have been published, a synthesized analysis focusing on the clinical characteristics, risk factors, and comparative effectiveness of antimicrobial management strategies for invasive *M. hominis* infections in adults is lacking.

Here, we report a case of postoperative *M. hominis* wound infection in an elderly male following orthopedic surgery. Furthermore, through a review of the literature, we aim to consolidate existing evidence to elucidate the predominant clinical features, identify factors associated with treatment outcomes, and evaluate therapeutic approaches. This synthesis intends to enhance clinical awareness, guide timely diagnosis, and inform optimal antimicrobial strategy for this rare but consequential pathogen.

## Case report

**Figure 1:**
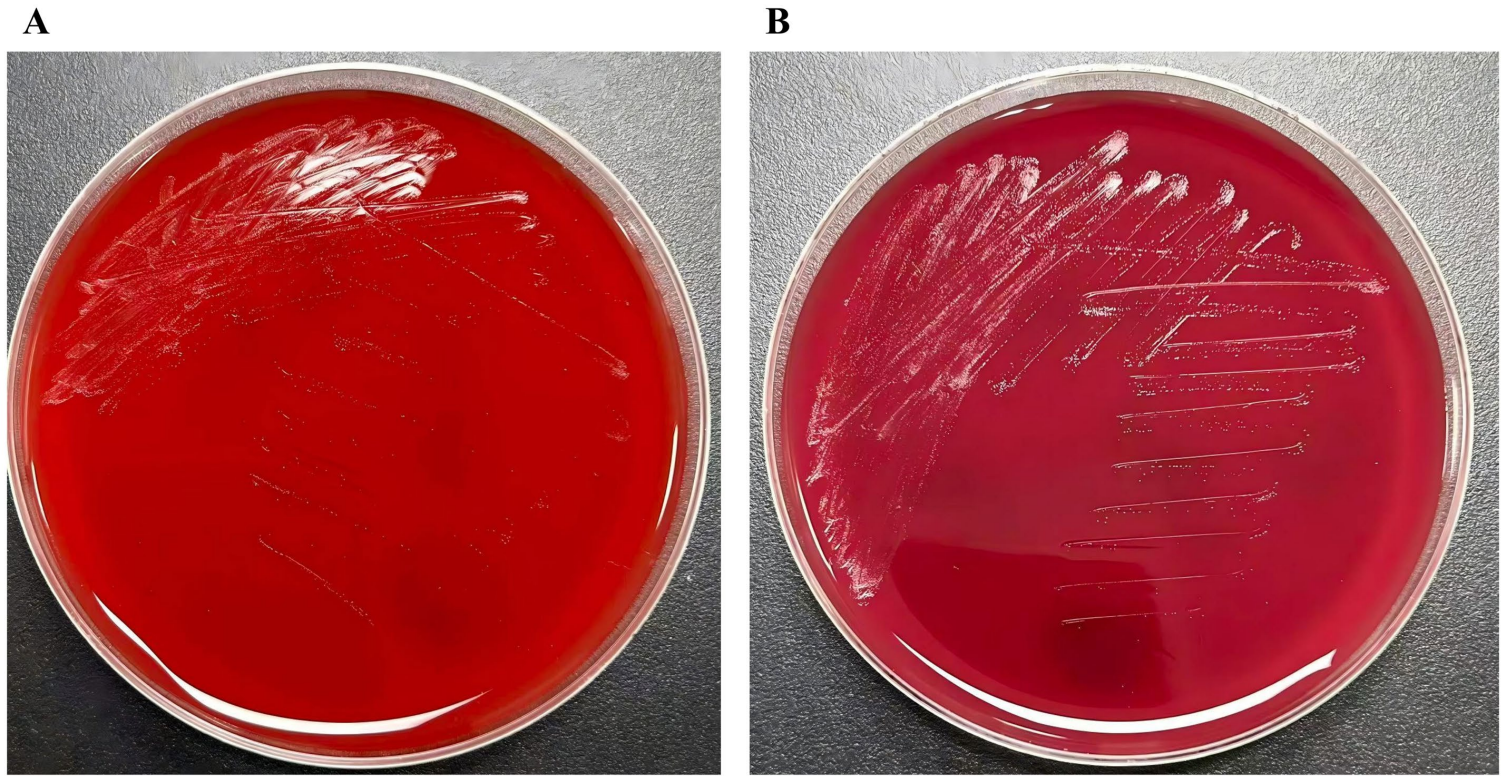
Culture results of *M. hominis* on Columbia blood agar. **(A)** After 3 days (72 h) of incubation on Columbia blood agar. **(B)** After 4 days (96 h) of incubation on Columbia blood agar.

**Figure 2:**
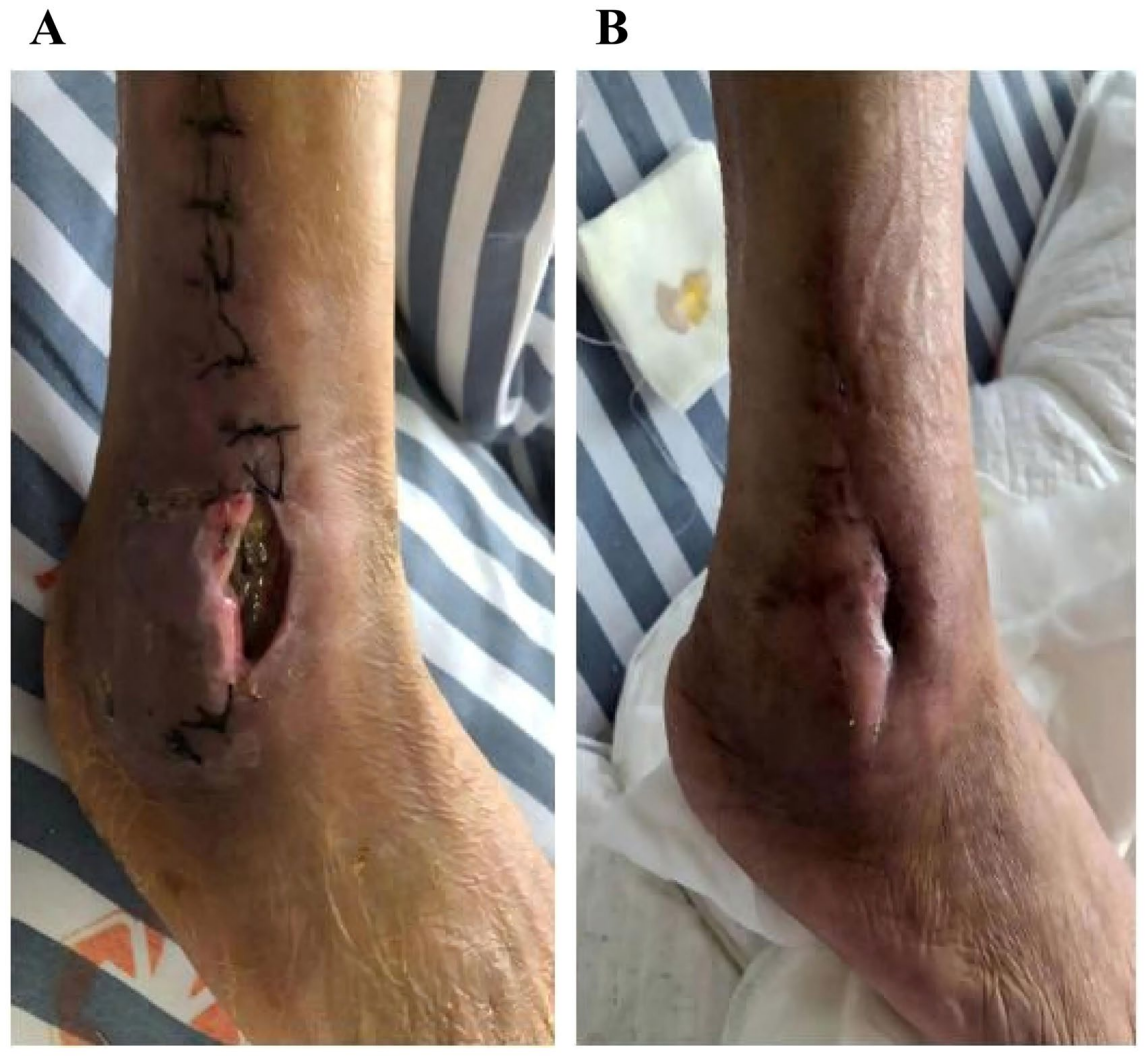
Wound healing process of the surgical site. **(A)** The surgical wound on April 20 (postoperative day 6) showing swelling, erythema, and increased exudate. **(B)** The surgical wound on May 27 (postoperative day 33) showing complete healing with no erythema or exudation, pain resolved, and restored mobility.

**Figure 3:**
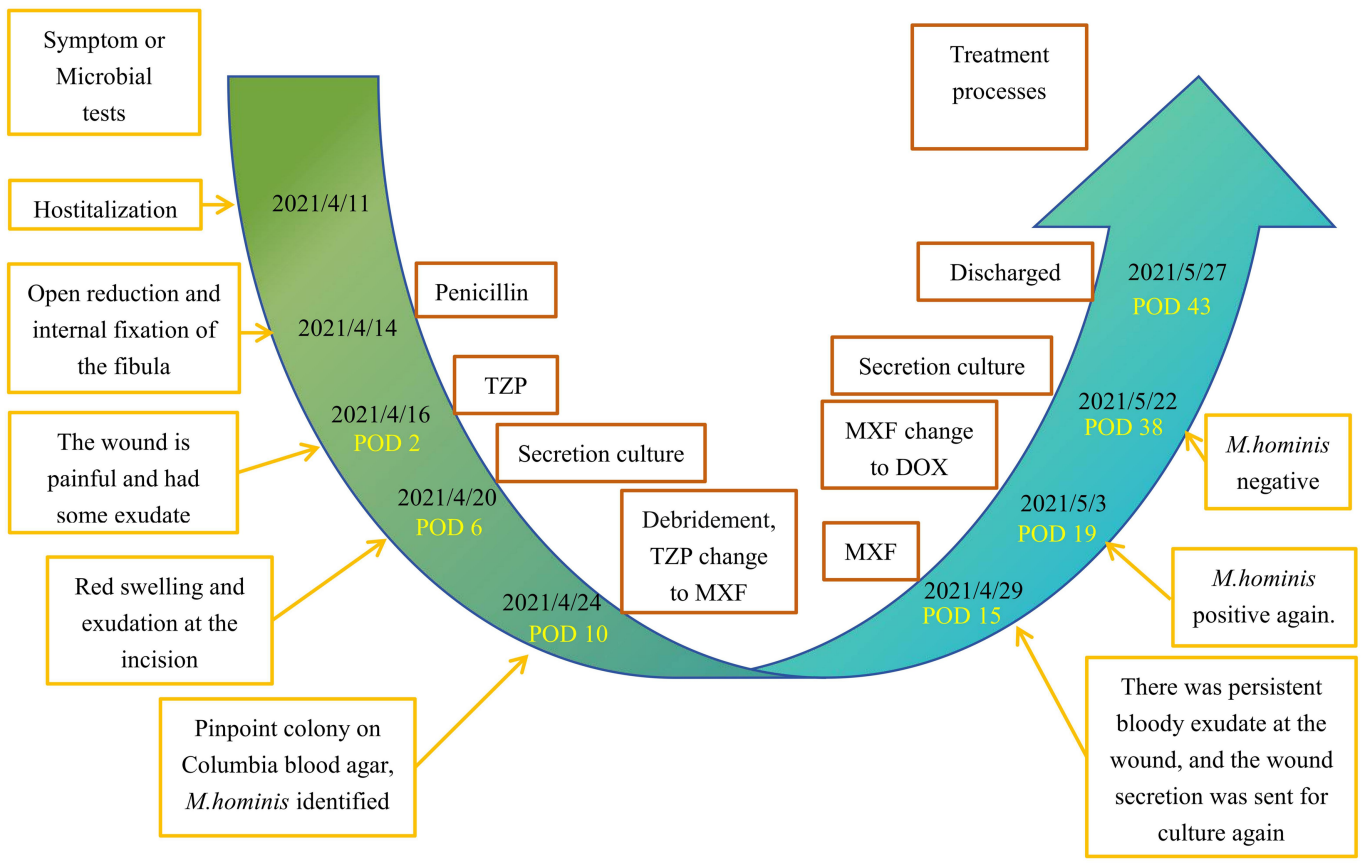
Timelines of patient’s diagnosis and treatment. TZP, piperacillin-tazobactam; MXF, moxifloxacin; DOX, doxycycline.

A 60-year-old previously healthy male was admitted 2 days after sustaining trauma that resulted in an open Gustilo-Anderson type IIIA fracture of the right distal fibula. Computed tomography (CT) revealed a comminuted fracture of the distal fibula accompanied by multiple fractures of the left ribs. Further imaging and laboratory assessment delineated the extent of his injuries and systemic involvement. Chest CT identified bilateral pleural effusions and dependent pulmonary consolidations. Abdominal CT demonstrated small, round hypodensities in the right hepatic lobe and multiple small, hyperdense nodules within the gallbladder, findings suggestive of hepatic cysts and cholelithiasis. Bilateral perinephric stranding was also observed, indicative of possible renal contusion. The patient complained of left-sided back pain and swelling with tenderness around the distal fibula. Notably, laboratory tests upon admission revealed impaired liver and renal function, with elevated levels of alanine aminotransferase (ALT 216.9 U/L), aspartate aminotransferase (AST 203.6 U/L), blood urea nitrogen (BUN 9.66 mmol/L), and creatinine (CREA 132.8 μmol/L), along with decreased total protein (TP 55.8 g/L) and albumin (ALB 30.9 g/L). Admission tests also revealed anemia (hemoglobin 87 g/L). On April 14, 2021, he underwent open reduction and internal fixation of the right fibula with ligament repair and bone grafting under general anesthesia. Postoperatively, prophylactic antimicrobial therapy with piperacillin–tazobactam (4.5 g intravenously every 8 h) was initiated. The patient had an indwelling urinary catheter for a cumulative duration of 6 days during the hospital stay. On April 20 (postoperative day 6), the surgical wound of the right fibula became markedly swollen and erythematous with increased exudate and tenderness, suggestive of poor wound healing. Purulent exudate (pus) was collected for microbiological culture on this day. Gram staining showed no visible bacteria, but numerous neutrophils were observed. The purulent sample was incubated at 35 °C in 5% CO_2_, and after 4 days, pinpoint, non-hemolytic colonies were noted on blood agar, with multiple pinpoint colonies also visible on Columbia blood agar (Figure 1). On April 24 (postoperative day 10), the laboratory reported the identification of these colonies by matrix-assisted laser desorption/ionization time-of-flight mass spectrometry (MALDI-TOF MS), which demonstrated a 99.9% match with *M. hominis*. No other bacterial growth was detected, and histopathological examination was not performed. Based on this initial culture report and empirical clinical judgment, the antimicrobial regimen was adjusted from piperacillin-tazobactam to moxifloxacin (400 mg intravenously once daily) on April 24. In addition, vacuum sealing drainage (VSD) with continuous irrigation and wound care was applied. The patient experienced slight symptomatic relief, but overall response was suboptimal. On May 3 (postoperative day 19), a second purulent sample culture was obtained, which again yielded *M. hominis*. Antimicrobial susceptibility testing was performed, and therapy was switched to doxycycline (100 mg orally every 12 h) for a total course of 3 weeks. By May 27 (postoperative day 33), the wound showed no erythema or exudation, pain had resolved, lower limb mobility was restored, and the surgical site had healed well. The patient was discharged in good condition (Figure 2). Throughout the clinical course, *M. hominis* was the only pathogen isolated from wound specimens, underscoring its definitive pathogenic role in this infection and guiding subsequent antimicrobial management. A timeline summarizing the patient’s clinical course, key microbiological events, and antibiotic therapy is presented in Figure 3.

### Microbiology

The purulent exudate (pus) from the distal fibula were inoculated onto Columbia blood agar (bioMérieux) and incubated at 35 °C in 5% CO_2_. A change in broth color was observed after 24–48 h of incubation. After 4 days of incubation, pinpoint, non-hemolytic colonies were observed (Figure 1). Colony identification was performed using matrix-assisted laser desorption/ionization time-of-flight mass spectrometry (MALDI-TOF MS; VITEK MS system, bioMérieux), which confirmed *Mycoplasma hominis* with a 99.9% match score. This identification was successfully repeated from a second sample obtained on May 3. Antimicrobial susceptibility testing was performed using a commercially available broth-based phenotypic susceptibility kit (urea-arginine LYO2 broth; Lizhu, Zhuhai, China) according to the manufacturer’s instructions. Testing was conducted under anaerobic conditions at 35 °C–37 °C for 48 h. Results were interpreted based on the interpretive criteria provided by the kit manufacturer: yellow indicated negative growth, while clear red indicated positive growth. The minimum inhibitory concentrations (MICs; μg/mL) were as follows: ciprofloxacin >2, ofloxacin >4, gatifloxacin >8, levofloxacin >4, erythromycin >4, azithromycin >4, clarithromycin >4, josamycin ≤2, roxithromycin >4, doxycycline ≤4, sparfloxacin >4, and minocycline ≤4. According to the interpretive criteria provided with the kit, the isolate was categorized as susceptible to doxycycline, josamycin, and minocycline.

### Therapy and outcome

At a 1-year follow-up, the patient demonstrated significant clinical improvement with complete wound healing. No evidence of recurrence or related complications was observed during this period.

## Literature review

In order to contextualize our case and summarize the existing evidence on invasive *M. hominis* infections in adults, we performed a narrative review of the literature. We conducted a systematic search of the PubMed database for English-language publications between January 1, 1980, and August 31, 2025. The search strategy employed the following Boolean operators: the search terms for the pathogen (“*Mycoplasma hominis*” OR “*M. hominis*”) were combined with AND to terms for infection sites (“wound infection” OR “sternal osteitis” OR “abscess” OR “mediastinitis” OR “pleuritis” OR “peritonitis” OR “encephalitis” OR “meningitis” OR “arthritis” OR “joint infection” OR “prosthetic joint infection”). We are aware of the recent taxonomic reclassification to *Metamycoplasma hominis*. A supplementary search of the database using this term did not retrieve any additional clinical case reports eligible for inclusion. This initial search yielded 80 citations.

We aimed to identify reports describing the clinical characteristics, management, and outcomes of invasive *M. hominis* infections in adult patients (≥18 years). Articles were initially screened by title and abstract by two authors (YL and FL) to identify potentially relevant reports of extragenital *M. hominis* infections. Full-text articles were then assessed for eligibility. We included case reports and case series that provided sufficient detail on patient demographics, infection site, treatment (antimicrobial regimen and duration), and clinical outcome (cure/failure or survival/death). We excluded studies focusing solely on neonatal infections, genitourinary tract infections without invasive disease, reviews without original case data, and reports where treatment details or outcomes were not clearly stated. Any discrepancies in study selection were resolved through discussion with a senior author (QZ or YC). This search and screening process yielded 80 potentially relevant articles, of which 44 articles describing 64 unique adult cases met our inclusion criteria. Given the predominance of case reports and the heterogeneity in reporting, a formal quality assessment or risk-of-bias tool was not applied, consistent with the nature of a narrative review of such literature. Data from these 44 articles, along with the present case (total *n* = 65), were extracted by one author and verified by another. Extracted variables included demographic characteristics (age, sex), underlying medical conditions, infection site, antimicrobial therapy (agents and duration), and clinical outcomes (7–50).

Statistical analysis was performed using SPSS software version 26. Categorical variables were analyzed using the chi-square test or Fisher’s exact test, while non-parametric tests (Mann–Whitney U test) were applied to continuous variables. Variables with a *p*-value ≤ 0.05 in univariate analysis were entered into a forward stepwise logistic regression model to identify independent factors associated with treatment failure. Statistical significance was defined as *p* ≤ 0.05. Age and duration of antimicrobial therapy were reported as median values with interquartile ranges (IQR).

## Results of the review

A total of 44 articles were identified, comprising 64 reported cases of *M. hominis* infection, with several articles describing multiple cases. Including the present case, 65 cases were analyzed (Table 1). The majority of patients were male (51/65, 78.5%), with a median age of 52 years (IQR: 34–63). Overall, 17 patients (26.2%) experienced treatment failure, defined as death attributable to the infection or clinical/microbiological relapse/persistence. Of these, 14 patients died and 3 experienced relapse. Notably, fatal outcomes were frequently observed in patients with invasive disease at sterile sites (e.g., CNS, mediastinum) or in the setting of post-transplantation immunosuppression and major comorbidities. Invasive *M. hominis* infection was more frequently observed in patients with a history of surgery or urinary catheterization (64/65, 98.5%), followed by those with cardiopulmonary insufficiency (42/65, 64.6%), immunosuppressive therapy (30/65, 46.2%), and post-transplantation status (25/65, 38.5%). The most common sites of infection were skin and bone (39/65, 60.0%), followed by pleural/peritoneal effusions (12/65, 18.5%) and the central nervous system (7/65, 10.8%). Among patients with skin/bone infections, 25/39 (64.1%) had underlying cardiopulmonary insufficiency. Of the 12 patients with pleural/peritoneal infections, 10 were post-transplant recipients, including four with idiopathic pulmonary fibrosis and three with pulmonary hypertension. Among seven patients with central nervous system infections, five had a history of trauma and three had hypertension. Meanwhile, surgical interventions (64, 98.5%) for source control (e.g., debridement, drainage, or hardware removal) were documented in a substantial proportion of cases, especially those involving deep-seated infections such as mediastinitis, osteomyelitis, and prosthetic joint infections.

**Table 1:** Clinical characteristics of 65 cases of *Mycoplasma hominis* infection.

Characteristics	All patient (*N* = 65)	Cured (*N* = 48)	Treatment failure (*N* = 17)	Univariate analysis	Multivariate analysis	
				*p*-value	*p*-value	OR (95% CI)
Sex						
Male	51	39	12	0.358	—	—
Female	14	9	5			
Age, years, median (Interquartile range)	52 (34–63)	54 (34.75–62.25)	46 (32.5–64)	0.988	—	—
Predominant underlying condition						
Trauma	12	11	1	0.233	—	—
Post-transplantation	25	14	11	0.010	0.044	6.045 (1.053–34.710)
Cardiopulmonary insufficiency	42	27	15	0.037	0.155	
Surgical operation	64	47	17	>0.999	—	—
Urinary catheter insertion	64	47	17	>0.999	—	—
Cerebrovascular disease	11	9	2	0.777	—	—
Hypertension	7	4	3	0.542	—	—
Corticosteroid pharmacotherapy	30	19	11	0.074	—	—
Infection site						
Skin/bone	39	30	9	0.489	—	—
Pleural and ascites	12	10	2	0.642	—	—
Brain	7	5	2	>0.999	—	—
Others	7	3	4	0.129	—	—
Antimicrobial treatment duration, days, median (Interquartile range)	30 (14–54.5)	42 (17.25–59)	17 (8.5–42)	0.017	0.004	0.089 (0.017–0.466)
Antimicrobial therapy						
Doxycycline-treated	40	31	9	0.397	—	—
Non-doxycycline-treated	25	17	8			

Doxycycline-treated: This group included doxycycline monotherapy, or doxycycline in combination with fluoroquinolones, clindamycin, or aminoglycosides. Non-doxycycline-treated: This group included any single-agent or combination therapy regimen excluding doxycycline.

Multivariate logistic regression revealed that prolonged antimicrobial therapy was a protective factor against mortality [*p* = 0.004, OR (95% CI): 0.089 (0.017–0.466)], whereas transplantation was an independent risk factor for death [*p* = 0.044, OR (95% CI): 6.045 (1.053–34.710)] (Table 1).

Regarding diagnostic methods, among the 65 cases, the reported methods for definitive identification of *M. hominis* included 16S rRNA gene sequencing (*n* = 13), matrix-assisted laser desorption/ionization time-of-flight mass spectrometry (MALDI-TOF MS) (*n* = 10), targeted PCR assays (*n* = 10), and metagenomic next-generation sequencing (mNGS) (*n* = 4). For the remaining cases, the specific identification method was not explicitly stated in the source reports.

Regarding treatment, 40 patients (61.5%, median age 54.5 years, median duration 42 d) received doxycycline-containing regimens (including doxycycline monotherapy or doxycycline combined with fluoroquinolones, clindamycin, or aminoglycosides), with 9 failures (22.5%). Twenty-five patients (38.5%, median age 48 years, median duration 14 d) received non-doxycycline regimens (any single or combination therapy excluding doxycycline), with 8 failures (32.0%) (Table 2). No significant differences were observed between the two groups in terms of age, sex, comorbidities, or overall outcome. Among the 48 successfully treated patients, further analysis revealed that in skin/bone infections, doxycycline-containing regimens had a longer duration (42 d) than non-doxycycline regimens (36.5 d). In pleural/peritoneal infections, doxycycline-containing regimens also required longer treatment courses (42 d vs. 24.5 d). The most common successful regimens were doxycycline combined with fluoroquinolones (17/48, 35.4%), doxycycline monotherapy (12/48, 25.0%), and fluoroquinolone monotherapy (10/48, 20.8%), with median durations of 42, 42, and 24.5 d, respectively. All of these regimens achieved favorable outcomes.

**Table 2:** Clinical characteristics of treatment with doxycycline vs. non-doxycycline.

Characteristics	Treated with doxycycline (*N* = 40)	Treated with non-doxycycline (*N* = 25)	*p*-value
Sex			
Male	33	18	0.316
Female	7	7	
Age, years, median (Interquartile range)	54.5 (38.5–59.75)	48 (26–67)	0.808
Predominant underlying condition			
Trauma	4	8	0.058
Post-transplantation	18	7	0.171
Cardiopulmonary insufficiency	29	13	0.093
Surgical operation	40	24	0.811
Urinary catheter insertion	40	24	0.811
Cerebrovascular disease	5	6	0.229
Hypertension	3	4	0.507
Corticosteroid pharmacotherapy	23	7	0.020
Infection site			
Skin/bone	27	12	0.118
Pleural and ascites	8	4	0.686
Brain	3	4	0.507
Others	2	5	0.137
Antimicrobial treatment duration, days, median (Interquartile range)	42 (28–56)	14 (11–36.5)	0.003
Therapeutic inefficacy	9	8	0.397

Treated with doxycycline: This group included doxycycline monotherapy, or doxycycline in combination with fluoroquinolones, clindamycin, or aminoglycosides. Treated with non-doxycycline: This group included any single-agent or combination therapy regimen excluding doxycycline.

## Discussion

*Mycoplasma hominis* is a fastidious pathogen capable of causing severe extragenital infections, particularly in immunocompromised or post-procedural patients (1, 11). Diagnosis is notoriously delayed due to its slow growth, invisibility on routine Gram stain, and resistance to empirical β-lactams, leading to increased morbidity and mortality (32). This literature review, encompassing 65 cases of invasive *M. hominis* infection, provides several novel insights that extend beyond individual case reports. First, it quantifies and highlights the exceptionally high burden of this infection among solid organ transplant recipients (38.5%) and patients following major cardiothoracic procedures, defining these as paramount risk groups. Second, through multivariate analysis of pooled case data, it identifies prolonged antimicrobial therapy duration as a stronger independent predictor of success than specific drug choice, while confirming post-transplantation status as a key risk factor for treatment failure. Third, by systematically synthesizing management approaches, it enables the formulation of integrated, evidence-informed recommendations covering diagnosis, combination therapy (antimicrobials plus source control), and individualized follow-up—a guidance framework previously lacking for this uncommon pathogen.

Consistent with prior reports (2, 16, 18, 32, 36, 51), our review identified a history of surgery or urinary catheterization as the most common predisposing factor, followed by cardiopulmonary insufficiency, immunosuppressive therapy, and post-transplantation status. A key finding was the high prevalence among transplant recipients (38.5%) and patients undergoing cardiothoracic procedures, underscoring these as particularly high-risk settings (2, 52). The urogenital tract likely serves as a reservoir, with hematogenous spread (48) or direct inoculation (53) leading to infections at sites of anatomical disruption. This study delineates distinct clinical patterns: skin/bone infections were linked to trauma or surgery; pleural/peritoneal effusions predominated in transplant recipients; and CNS involvement was associated with neurosurgery or head trauma.

In our case, for example, the diagnosis was confirmed only after 4 days of incubation, during which the patient received ineffective therapy. This highlights that a high index of suspicion is necessary in post-procedural or immunocompromised patients with culture-negative but clinically suggestive infections. Early clinical recognition and timely initiation of effective antimicrobial therapy are therefore essential. The presented case underscores that invasive *M. hominis* infection often occurs within a complex clinical tableau, extending beyond a localized wound. Our patient was an elderly male with significant polytrauma, including the open fracture, multiple rib fractures, and imaging findings consistent with pulmonary contusion/consolidation, pleural effusions, and possible renal contusion. Additionally, pre-existing conditions such as hepatic cysts and cholelithiasis were identified. This context provides a plausible, multifactorial explanation for the observed systemic laboratory abnormalities—including elevated liver enzymes, impaired renal function, anemia, and hypoalbuminemia. These derangements likely resulted from the combined effects of direct traumatic organ injury, the systemic inflammatory response to severe infection, and underlying comorbidities. Critically, this patient was not a transplant recipient, highlighting that severe organ dysfunction in such settings can arise independently of immunosuppression, primarily driven by major physiological stress and anatomical disruption. This comprehensive profile reinforces the importance of evaluating and supporting concurrent organ systems when managing invasive *M. hominis* infections.

The intrinsic resistance of *M. hominis* to cell-wall-active agents necessitates alternative regimens. While our isolate and published data (14) show susceptibility to tetracyclines (doxycycline, minocycline), and a systematic review suggests that tetracycline resistance remains uncommon in clinical isolates (54), and variable activity against fluoroquinolones, our multivariate analysis revealed that prolonged antimicrobial therapy duration was a stronger protective factor against failure than the specific drug class. Successful regimens, often doxycycline-based (monotherapy or combination), had a median duration of 42 days, especially for deep-seated infections. Conversely, post-transplantation status independently increased the risk of treatment failure, highlighting the compounded challenge of managing infection in the context of iatrogenic immunosuppression.

Based on our synthesis, we propose a structured approach to managing suspected invasive *M. hominis* infection. In high-risk patients (post-transplant, major cardiothoracic/orthopedic/neurosurgery) with persistent fever, negative routine cultures, and poor response to broad-spectrum antibiotics, *M. hominis* should be suspected. Diagnostic efforts must be proactive: wound/sterile-site specimens should be submitted for prolonged culture on enriched media (e.g., blood agar) with explicit request for mycoplasma consideration. Parallel use of rapid diagnostic tools—MALDI-TOF MS or specific PCR—is critical to shorten the diagnostic window, as demonstrated in our case and supported by its validation for species-level identification when appropriate spectral libraries are available (2, 26–28, 32). Molecular methods such as PCR and 16S rRNA sequencing can serve as important adjuncts (2, 26–28, 32), and combining classical microbiology with molecular techniques represents the most reliable strategy for establishing a diagnosis in complex cases. Our review of 65 cases confirms that diagnosis often relies on advanced techniques: 16S rRNA sequencing, MALDI-TOF MS, and PCR were the most frequently reported definitive methods. This pattern underscores the frequent failure of routine diagnostic pathways and reinforces the need for proactive, specific testing when *M. hominis* is suspected.

In addition to pharmacological therapy, prompt source control proved essential for favorable outcomes. Timely removal of catheters, drainage of effusions, and surgical debridement or reconstruction, when necessary, were critical for favorable outcomes. Our review suggests that effective source control, though often delayed by diagnostic challenges, is a key determinant of success, particularly in deep-seated or device-associated infections. Effective management thus rests on the combination of timely antimicrobial therapy and source control. Doxycycline, combined with a fluoroquinolone such as moxifloxacin or levofloxacin where susceptibility is confirmed, represents a preferred empirical and targeted regimen. Therapy duration should be prolonged, often extending to 6–8 weeks or longer for complex infections (mediastinitis, osteomyelitis, device-associated), with duration guided by clinical response, infection site, and host immune status. Because *M. hominis* grows slowly and may persist in biofilm-like niches, inadequate source control likely contributes to recurrence and treatment failure.

Given these diagnostic challenges, the role of microbiological follow-up cultures in managing *M. hominis* infection warrants clarification. During the diagnostic and initial treatment phase, repeat cultures are often critical. They can confirm the diagnosis when initial tests are equivocal and, as demonstrated in our case, provide definitive evidence of persistent infection despite empiric therapy, thereby guiding essential antimicrobial changes. After completion of targeted therapy, the necessity of a routine clearance culture is less definitive and should be individualized. Such cultures are most warranted in patients with persistent clinical signs (e.g., ongoing drainage, fever), incomplete radiographic resolution, retained prosthetic material, or profound immunosuppression. In contrast, for patients who exhibit clear clinical and radiographic cure, sustained clinical recovery is a more practical and sufficient endpoint, and invasive sampling solely for microbiological documentation may not be justified.

This study has limitations inherent to the literature review of case reports and case series. First, publication bias is a significant concern; the true incidence of invasive *M. hominis* infection is likely underestimated. While clinical reports of extragenital infections are increasing—often associated with transplantation/immunosuppression and major surgery—many cases likely remain undiagnosed or unreported due to the pathogen’s fastidious nature, resistance to empirical *β*-lactams, and variability in diagnostic workflows. Consequently, the 64 cases analyzed here represent only a reported subset of the probable clinical burden. Second, heterogeneous reporting across included cases limits causal inference. Furthermore, the impact of specific adjunctive surgical interventions (e.g., debridement, drainage) on outcomes could not be analyzed due to inconsistent reporting of these details. Meanwhile, the antimicrobial susceptibility testing for the presented case relied on a commercial phenotypic kit. While clinically useful for guiding therapy, this method provides a susceptibility categorization rather than precise minimum inhibitory concentration values, which represents a methodological limitation compared to standardized broth microdilution. Additionally, standardized long-term follow-up data beyond the immediate hospitalization period were scarce, limiting assessment of definitive cure rates and long-term sequelae. Nonetheless, this review and pooled analysis revealed consistent patterns that can inform clinical practice. Despite these limitations, our analysis consolidates existing evidence and provides a practical, evidence-informed framework for the diagnosis and management of invasive *M. hominis* infections. Early recognition in high-risk settings, aggressive diagnostic pursuit, a combination of prolonged targeted antibiotics and timely source control, and individualized follow-up are paramount to improving patient outcomes.

## Conclusion

Our review of the literature concludes that although rare, *M. hominis* can cause severe postoperative and extragenital infections, most commonly involving the skin and bone, mediastinum, pleural/peritoneal cavities, and the central nervous system. Owing to difficulties in culture, delayed diagnosis, and intrinsic resistance to standard empirical antibiotics, clinical outcomes are often poor and the true incidence may be underestimated. Our review indicates that these infections are more frequently observed in patients with surgical history, urinary catheterization, cardiopulmonary insufficiency, immunosuppression, or post-transplantation status. Prolonged antimicrobial therapy was associated with improved outcomes, whereas post-transplantation status carried a higher risk of treatment failure. Given the limited therapeutic options, heightened clinical vigilance, early application of advanced diagnostic tools, adequate courses of active antimicrobial agents, and appropriate surgical interventions remain essential to improve patient prognosis.

## Data Availability

The raw data supporting the conclusions of this article will be made available by the authors, without undue reservation.
